# miR-26a desensitizes non-small cell lung cancer cells to tyrosine kinase inhibitors by targeting PTPN13

**DOI:** 10.18632/oncotarget.9920

**Published:** 2016-06-07

**Authors:** Shudi Xu, Tao Wang, Zhiwei Yang, Ying Li, Weijie Li, Ting Wang, Shan Wang, Lintao Jia, Shengli Zhang, Shengqing Li

**Affiliations:** ^1^ Department of Respiratory Medicine, Huashan Hospital, Fudan University, Shanghai, China; ^2^ Department of Respiratory Medicine, 9th Hospital of Xi'an, Xi'an, China; ^3^ Department of Neurology, Shaanxi Provincial People's Hospital, Xi'an, China; ^4^ Department of Applied Physics, Xi'an Jiaotong University, Xi'an, China; ^5^ Department of Respiratory Medicine, Shaanxi Provincial Second People's Hospital, Xi'an, China; ^6^ Department of Respiratory Medicine, Shaanxi Provincial People's Hospital, Xi'an, China; ^7^ Department of Biochemistry and Molecular Biology, Fourth Military Medical University, Xi'an, China

**Keywords:** epidermal growth factor receptor, tyrosine kinase inhibitor, non-small cell lung cancer, miR-26a, protein tyrosine phosphatase non-receptor type 13

## Abstract

Epidermal growth factor receptor (EGFR)-targeted tyrosine kinase inhibitors (TKIs) have emerged as first-line drugs for non-small cell lung cancers (NSCLCs). However, the resistance to TKIs represents the key limitation for their therapeutic efficacy. We found that miR-26a was upregulated in gefitinib-refractory NSCLCs; miR-26a is downstream of EGFR signaling and directly targets and silences protein tyrosine phosphatase non-receptor type 13 (PTPN13) to maintain the activation of Src, a dephosphorylation substrate of PTPN13, thus reinforcing EGFR pathway in a regulatory circuit. miR-26a inhibition significantly improved NSCLC responses to gefitinib. These data revealed a novel mechanism of NSCLC resistance to TKI treatment.

## INTRODUCTION

Genetic variations leading to overexpression or mutation of the epidermal growth factor receptor (EGFR) play essential roles in the occurrence and progression of lung cancers including non-small cell lung cancers (NSCLCs) [1–2]. Fortunately, a cohort of tyrosine kinase inhibitors (TKIs) targeting to activated EGFR have been approved for standard NSCLC therapy and have provided great benefits for patients [3]. However, primary or acquired drug resistance has become a formidable barrier to the success of TKI therapy of NSCLCs [4]. Resistance of neoplastic cells to TKIs arises from diverse alterations resulting in abolished TKI binding to EGFR, constitutive downstream kinase activity independent of EGFR [5], or activation of alternative growth factor signaling such as human epidermal growth factor receptor (HER) family, hepatocyte growth factor (HGF)/c-Met or insulin-like growth factor 1 receptor (IGF1R) pathways [4, 6–7]. Nevertheless, the mechanisms underlying TKI resistance of NSCLCs remain to be extensively understood [7].

microRNAs (miRNAs) have been well documented to post-transcriptionally silence specific genes, thus exerting wide regulation on cell behaviors including transformation and drug resistance [8–9]. According to their distinct roles in carcinogenesis, miRNAs have been classified as oncogenic and tumor suppressor miRNAs [8]. However, the involvement of individual miRNAs in the development and progression of cancers can be complicated [8, 10]. In this respect, miR-26a was found to antagonize carcinogenesis in the mammary, prostate, pancreas and stomach by targeting the Polycomb group protein EZH2, myeloid cell leukemia 1 (Mcl-1) or metadherin, cyclin E2, and fibroblast growth factor 9 (FGF9), respectively [11–16]; miR-26a suppression of melanoma, prostate and liver cancers was also attributed to its role in enhancing miRNA biogenesis by targeting Lin28B and Zcchc11 [17]. In contrast, miR-26a was established to target the tumor suppressor PTEN in various types of cells, whereby it facilitates the development of leukemia, glioblastoma and lung cancer [18–20]. Here, we found that miR-26a is highly expressed in TKI-resistant NSCLC cells, and that miR-26a promotes the proliferation and confers TKI resistance of lung cancer cells by inhibiting protein tyrosine phosphatase non-receptor type 13 (PTPN13); PTPN13 downregulation increases Src activation and enhances EGFR downstream signaling. The upregulation of miR-26a by EGFR pathway was also observed in NSCLC cells.

## RESULTS

### miR-26a is overexpressed in lung cancer cells resistant to tyrosine kinase inhibitor

**Figure 1:**
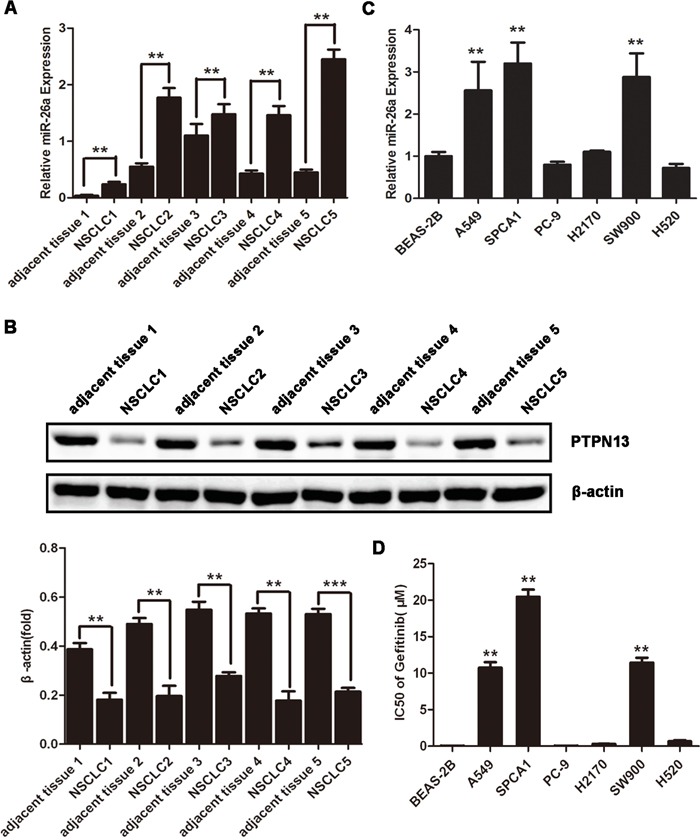
miR-26a is highly expressed in TKI-resistant NSCLC cells **A.** qRT-PCR assays of NSCLC samples and paired adjacent normal tissues. Data are represented as the mean ± SD of three replicates. **P<0.01 *vs* adjacent tissue. **B.** Western blot assays of NSCLC samples and the paired adjacent normal tissues using anti-PTPN13 antibody. Data are representative of 3 independent experiments. All the protein levels measured with densitometry and normalized to β-actin. Each bar represents the mean±SD from three experiments. **p<0.01*vs* control. **C.** qRT-PCR assays of the indicated human NSCLC cell lines or immortalized bronchial epithelial cell line, BEAS-2B. Data are represented as the mean ± SD of three replicates. *P<0.05, **P<0.01 *vs* BEAS-2B cells. **D.** NSCLC or immortalized bronchial epithelial cell lines were treated with or without gefitinib, and the doses of gefitinib that suppress cell growth by 50% (IC50) in MTT assays were plotted. Data are represented as the mean ± SD of three replicates. **P<0.01 *vs* BEAS-2B cells.

miR-26a has been well documented as either an oncogenic or tumor suppressor miRNA in various cancers [12, 14, 18–19]. We examined the expression of miR-26a in 5 non-small cell lung cancer (NSCLC) tissue samples, which were all EGFR-TKI resistant lung adenocarcinoma. Compared with the paired adjacent normal tissues, lung adenocarcinomas displayed significantly upregulated miR-26a expression, suggesting the involvement of miR-26a in the development of EGFR-TKI resistance in NSCLCs (Figure 1A). According to the screening results of miR-26a target genes using online miRNA target prediction tools, PTPN13, a phosphatase, may be a candidate target of miR-26a. Western blot study found that the expression level of PTPN13 decreased significanly in all cancer tissues compared with the paired adjacent normal tissues, suggesting that miR-26a may negatively regulate PTPN13 expression (Figure 1B). The NSCLC cell lines A549, SPCA1 and PC-9 are defined as lung adenocarcinomas, and H2170, SW900 and H520 as squamous cell carcinomas by American Type Culture Collection (ATCC). The NSCLC cell lines A549, SPCA1 and SW900 express higher levels of miR-26a than an immortalized human bronchial epithelial cell line, BEAS-2B (Figure 1C). Further study found that A549, SPCA1 and SW900 cell lines are more resistant to gefitinib than those with low levels of miR-26a (Figure 1D). We found a phenomenon that the higher level of miR-26a, the more resistance of some NSCLC cell lines to EGFR-TKIs. Therefore, miR-26a may be related to EGFR-TKIs resistance of NSCLCs.

### miR-26a promotes TKI resistance of lung cancer cells

**Figure 2:**
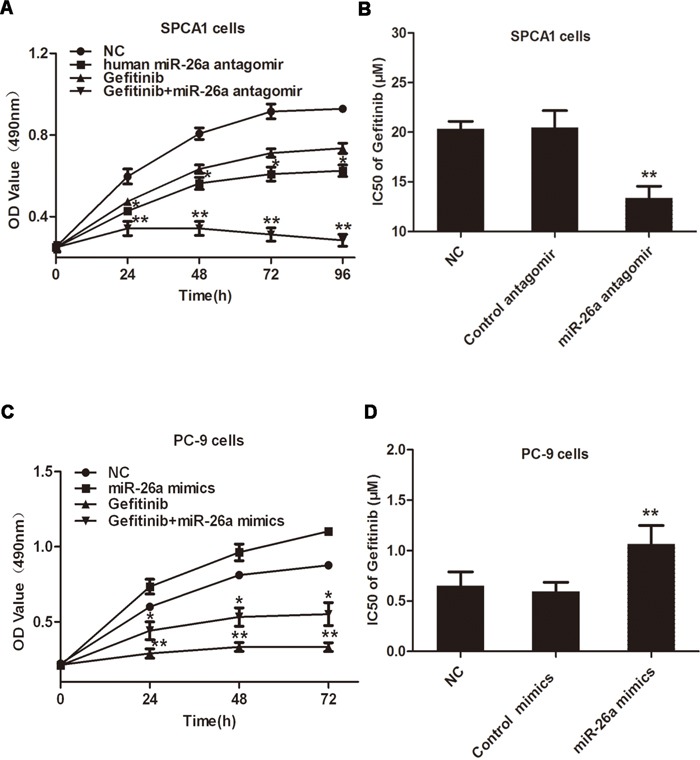
miR-26a desensitizes NSCLC cells to Gefitinib **A.** Lung cancer SPCA1 cells were transfected with miR-26a antagomirs, followed by treatment with or without 15 μM Gefitinib, MTT assays were performed at indicated time points. **B.** SPCA1 cells were transfected with miR-26a antagomirs or control antagomirs, followed by treatment with increasing concentrations of gefitinib (0-40μM) to measure the IC50 values. MTT assays were performed 48 h after Gefitinib treatment. **C.** Lung cancer PC-9 cells were transfected with miR-26a mimics, followed by treatment with or without 15 μM gefitinib, MTT assays were performed at indicated time points. **D.** PC-9 cells were transfected with miR-26a mimics or control mimics, followed by treatment with increasing concentrations of gefitinib to measure the IC50 values. MTT assays were performed 48 h after gefitinib treatment. Data are represented as the mean ± SD of three replicates. *P<0.05, ** *P*<0.01, *vs* NC group.

To test the involvement of miR-26a in regulating the malignant phenotype of NSCLCs, we modified miR-26a expression in cancer cells with distinct responsiveness to TKIs. SPCA1 cells with wild type EGFR and k-ras and high expression of miR-26a are EGFR-TKI resistant, and PC-9 cells with sensitive EGFR mutant (Del 746-750) and low levels of miR-26a are EGFR-TKI sensitive. As a result, transfection of TKI-resistant SPCA1 cells with miR-26a inhibitors caused dramatically suppressed cell growth or proliferation, and significantly improved the sensitivity of cells to gefitinib (Figure 2A and 2B). Conversely, introduction of synthesized miR-26a to PC-9, a known TKI-sensitive NSCLC cell line, resulted in accelerated cell growth and conferred resistance of cells to gefitinib (Figure 2C and 2D). These data suggest that miR-26a directly regulates the growth and TKI responsiveness of some lung adenocarcinomas.

### PTPN13 is a direct target of miR-26a in lung cancer cells

**Figure 3:**
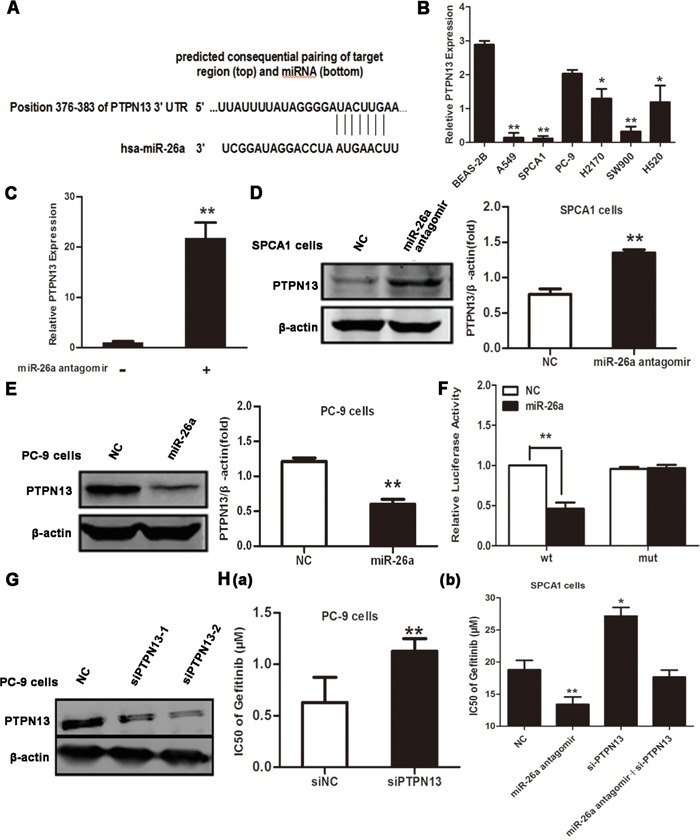
PTPN13 is targeted by miR-26a in NSCLC cells **A.** Schematic representation of predicted human miR-26a binding site at nucleotides 376−382 of the 3′ UTR of PTPN13 mRNA. The sequence of the mutated 3′ UTR in the corresponding site was also shown. **B.** qRT-PCR assays of PTPN13 expression in the indicated human NSCLC cell lines or immortalized bronchial epithelial cell line, BEAS-2B. Data are represented as the mean ± SD of three replicates. *P<0.05, ** *P*<0.01, *vs* BEAS-2B cells. **C.** qRT-PCR assays of PTPN13 expression in SPCA1 cells after transfection with control or miR-26a antagomirs. Data are represented as the mean ± SD of three replicates. ** *P*<0.01, *vs* control group. **D.** Western blot assays of PTPN13 expression in SPCA1 cells after transfection with control or miR-26a antagomirs. **E.** Western blot assay of PTPN13 expression in PC-9 cells after transfection with control or miR-26a mimics. **F.** Luciferase activity measured 24 h after co-transfection of HEK293 cells with a miR-26a mimic and a pGL3 construct containing wild-type or mutant PTPN13 3′UTR regions encompassing the predicted binding site of miR-26a as described in (A). Data were normalized to the luciferase activity of control (vehicle-transfected) cells, and are represented as the mean ± SD of three replicates. ** *P*<0.01, *vs* NC group. **G.** Western blot assays of PC-9 cells after transfection with control or PTPN13 targeted siRNAs. **H.** The IC50 values of gefitinib for PC-9 cells (a) transfected with PTPN13 siRNA (siPTPN13-2) were measured and plotted as described in Figure 2B and 2D. The IC50 values of gefitinib for SPCA1 cells (b) transfected with PTPN13 siRNA (siPTPN13-2) and/or miR-26a antagomirs. Data are represented as the mean ± SD of three replicates. *P<0.05, ** *P*<0.01, *vs* control group. In westrern blot, data are representative of 3 independent experiments. All the protein levels measured with densitometry and normalized to β-actin. Each bar represents the mean±SD from three experiments. **p<0.01*vs* control.

miRNAs exert a regulatory role on cell behaviors by post-transcriptionally silencing specific genes [8]. Therefore, we next searched for potential genes targeted by miR-26a in NSCLC cells. According to online miRNA target prediction tools, PTPN13 has the target sequence of miR-26a (Figure 3A). Indeed, the expression level of PTPN13 was much lower in EGFR-TKIs resistant lung cancer cell lines than BEAS-2B cells (Figure 3B), however EGFR-TKIs sensitive cells have relatively high expression levels of PTPN13 (Figure 3B). Transfection of miR-26a antagomir in SPCA1 cells caused a notable increase in cellular PTPN13 both at mRNA and protein levels (Figure 3C and 3D). In contrast, introduction of synthesized miR-26a into PC-9 cells significantly downregulated PTPN13 at protein levels(Figure 3E). We next constructed the reporter plasmid harboring the luciferase expression cassette flanked by the intact 3′ untranslated region (UTR) of PTPN13 mRNA or by the PTPN13 3′ UTR in which the putative miR-26a binding site was mutated. After cotransfection of HEK293 cells with the luciferase reporter constructs and synthesized miR-26a or control miRNA mimics, we found that miR-26a significantly suppressed luciferase expression from the construct containing the wild-type but not mutated PTPN13 3′ UTR (Figure 3F). To test whether PTPN13 is a functional target of miR-26a in determining gefitinib responsiveness of lung cancer cells, we silenced PTPN13 in PC-9 cells (Figure 3G). As a result, PTPN13 knockdown desensitized PC-9 cells to gefitinib(Figure 3H(a)) and at least partially abolished miR-26a antagomir-elicited sensitization of SPCA-1 cells to gefitinib (Figure 3H(b)). Therefore, PTPN13 is directly targeted and silenced by miR-26a to confer TKI resistance of NSCLC cells.

### PTPN13 can bind with and dephosphorylate Src kinase

**Figure 4:**
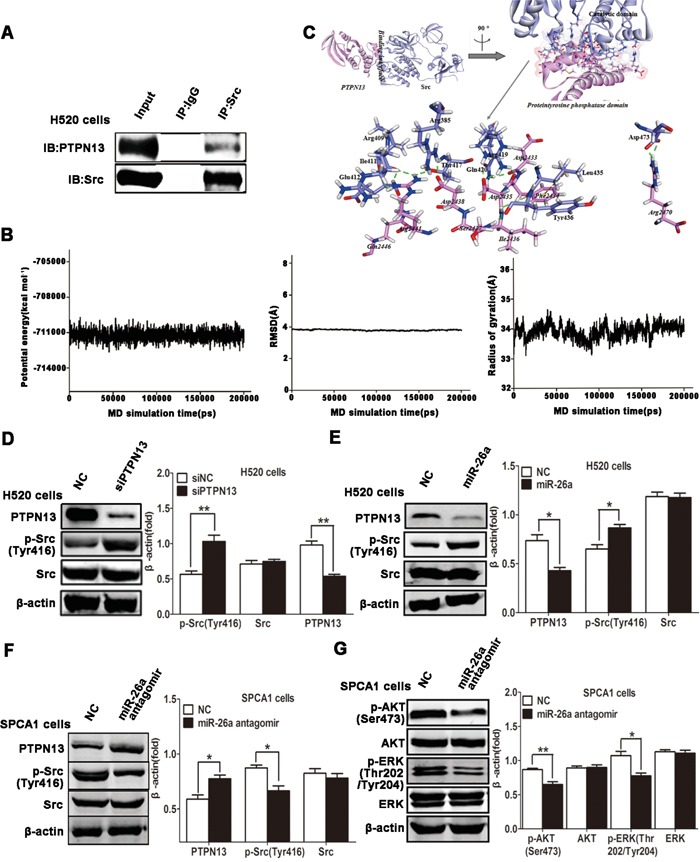
miR-26a/PTPN13 regulates EGFR/Src signaling **A.** Immunoprecipitation (IP) of PTPN13 with anti-Src antibody in H520 cells. The total protein for IP is 500μg, and the input protein is 30μg. **B.** The time-evolution potential energy, backbone-atom RMSD and radius of gyration for the PTPN13-Src^pTyr416^ complex. **C.** The propeller structure and key residues within the binding interface of PTPN13-Src^pTyr416^ complex. The Connolly surface of binding interface is colored by electrostatic potential. The key residues of PTPN13 and Src^pTyr416^ are represented by stick models. The C atoms are colored in cyan and pink for PTPN13 and Src^pTyr416^, and the H, N and O atoms are colored in white, blue and red, respectively. H-bonds and hydrophobic-interactions are labeled in dashed green and brown lines, respectively. **D.** Western blot assays of H520 cells after treatment with EGF (10 ng/ml) and transfected with control siRNA and PTPN13 siRNA. **E.** Western blot assays of H520 cells after treatment with EGF (10 ng/ml) and transfected with control or miR-26a mimics. **F, G.** Western blot assays of SPCA1 cells after treatment with EGF (10 ng/ml) and transfected with control or miR-26a antagomirs. In western blot, data are representative of 3 independent experiments. All the protein levels measured with densitometry and normalized to β-actin. Each bar represents the mean±SD from three experiments.*p<0.05, **p<0.01*vs* control group.

PTPN13 (NP_542416.1, ~2490 aa), is a multi-module containing phosphatase [32], with mediate associations with many cellular proteins in several steps of tumor progression. A protein tyrosine phosphatase domain (residues 2087-2485) is at its C terminus, and other regions (like PDZ domains) of PTPN13 are non-catalytic modules, with the activity of which regulates its cellular localization and/or interaction with substrates [21, 33–34]. Src kinase activation is necessary for lung cancer cell growth and survival [35–36]. Previous studies reveal that PTPN13 probably mediates regulation of cell homeostasis through PI3-kinase-dependent signaling pathways [37], and suppresses tumor aggressiveness through the inactivation of Src kinase (dephosphorylation) [38]. In this study, co-immunoprecipitation was used to study the relationship of PTPN13 and Src, and it was found that Src can bind with PTPN13 in H520 cells (Figure 4A). All members of Src kinase family have a catalytic domain preceded by two Src-homology domains (SH2 and SH3), including proto-oncogene tyrosine-protein kinase (NP_114183.1, ~542 aa) [22, 39]. SH2 and SH3 modules cooperate in regulating the phosphorylation activity of Src kinases [27, 40]. Numerous critical questions about the molecular determinants underlying the binding specificity of Src to PTPN13 remain unanswered. Here, modeling studies provide the first molecular description of the detailed interaction profiles of PTPN13 with Src.

The two generated models (PTPN13 and Src^pTyr416^) are all well-behaved during the 100-ns MD simulations, oscillating with minor variations and good stereochemical features (Figure S1-S4). It is consistent with previous MD simulations of PDZ domain protein [41] and Src kinases [22]. Therefore, the equilibrium of these simulations is reliable, and the stable structures are reasonable. The PTPN13-Src^pTyr416^ complex arrives to the equilibrium state since ~150 ns, with the RDOCK scoring value (Z_RDock) of −20.07 kcal .mol^−1^ (Figure 4B). The binding of PTPN13 with Src^pTyr416^ is characterized by the strong H-bonding interaction networks involving residues PTPN13:Asp2435, Arg2441, Arg2470, and Src^pTyr416^:Arg419, Arg409, Asp473, with the formation of five H-bonds (Table 1 and Figure 4C). There are other eight H-bonds, to further stable the bound complex (Table 1). Additionally, modest hydrophobic interactions exist between PTPN13 and Src^pTyr416^, such as residues PTPN13:Phe2434 and Src^pTyr416^:Leu435, with the closest atom distance of 5.09 Å (Figure 4C). Within the interfacial surfaces, polar residues Asp and Arg are likely to appear at the binding interface (13.89 and 16.67 %), with significant binding contributions (Table 1 and Figure 4C). In accord with our binding mode, the catalytic domain (residues 266-542) of Src, which includes phosphorylation site Tyr416, is covered by proteintyrosine phosphatase domain of PTPN13 (Figure 4C). Combined with the results of immunoprecipitation, regulation of Src activity by PTPN13 might be achieved through the direct hide of its catalytic domain and dephosphorylation of pTyr416, where could ordinarily be regulated by SH2 and SH3 binding modules [42]. The modeling results may contribute to intensively understanding on the biological role of PTPN13, as the first phosphatase able to inhibit Src through direct dephosphorylation in cancer cells.

**Table 1:** H-bonding interactions involved with residues of PTPN13 and Src^pTyr416^

Receptor (PTPN13)	Ligand (Src^pTyr416^)	Interaction constituents (Donor→ Acceptor)	Distance (Å)	Angle (°)
Asp2433	Gln420	Gln420:HE21 - Asp2433:O	1.98	139.9
Phe2434	Arg419	Arg419:HH21 - Phe2434:O	1.84	137.1
Asp2435	Arg419	Arg419:HH12 - Asp2435:OD2	1.89	146.9
Asp2435	Arg419	Arg419:HH22 - Asp2435:OD2	1.88	147.9
Ile2436	Leu435	Ile2436:HN - Leu435:O	3.06	93.2
Ser2437	Tyr436	Ser2437:HN - Tyr436:O	1.86	160.1
Asp2438	Arg385	Arg385:HH22 - Asp2438:OD2	1.84	148.1
Arg2441	Arg409	Arg2441:HH11 - Arg409:O	2.57	93.0
Arg2441	Ile411	Arg2441:HH12 - Ile411:O	1.76	146.5
Arg2441	Ile411	Arg2441:HH22 - Ile411:O	2.01	133.6
Arg2441	Thr417	Arg2441:HH22 - Thr417:OG1	2.27	118.3
Gln2446	Glu412	Gln2446:HE21 - Glu412:O	1.94	142.7
Arg2470	Asp473	Arg2470:HH12 - Asp473:OD2	1.89	158.5
Arg2470	Asp473	Arg2470:HH22 - Asp473:OD1	1.89	144.5

### miR-26a and PTPN13 are involved in mediating and regulating EGFR signaling

To Study the molecular mechanism of miR-26a involvement in Src signaling pathway, we next examined whether Src activity was affected by miR-26a in NSCLC cells. Indeed, knockdown of PTPN13 facilitated EGF-triggered activation of phospho-Src in H520 cells (Figure 4D). Introduction of miR-26a mimics into H520 cells enhanced Src phosphorylation by downregulating PTPN13 (Figure 4E). Conversely, transfection of SPCA-1 cells with miR-26a antagomirs caused a significant increase of PTPN13 expression and a dramatic suppression of Src phosphorylation in EGF-stimulated SPCA1 cells (Figure 4F). These data suggest that miR-26a maintains Src activation by silencing PTPN13 in lung carcinoma cells. Considering the interaction and reciprocal activation between Src and EGFR, we next investigated whether miR-26a reinforced EGFR signaling. Suppression of miR-26a resulted in a decrease in EGF-triggered phosphorylation and activation of AKT and ERK, both of which represent the canonical signal pathways downstream of EGF/EGFR (Figure 4G). Therefore, miR-26a promotes EGFR/Src/AKT/ERK signaling by targeting and silencing PTPN13 in NSCLC cells.

**Figure 5:**
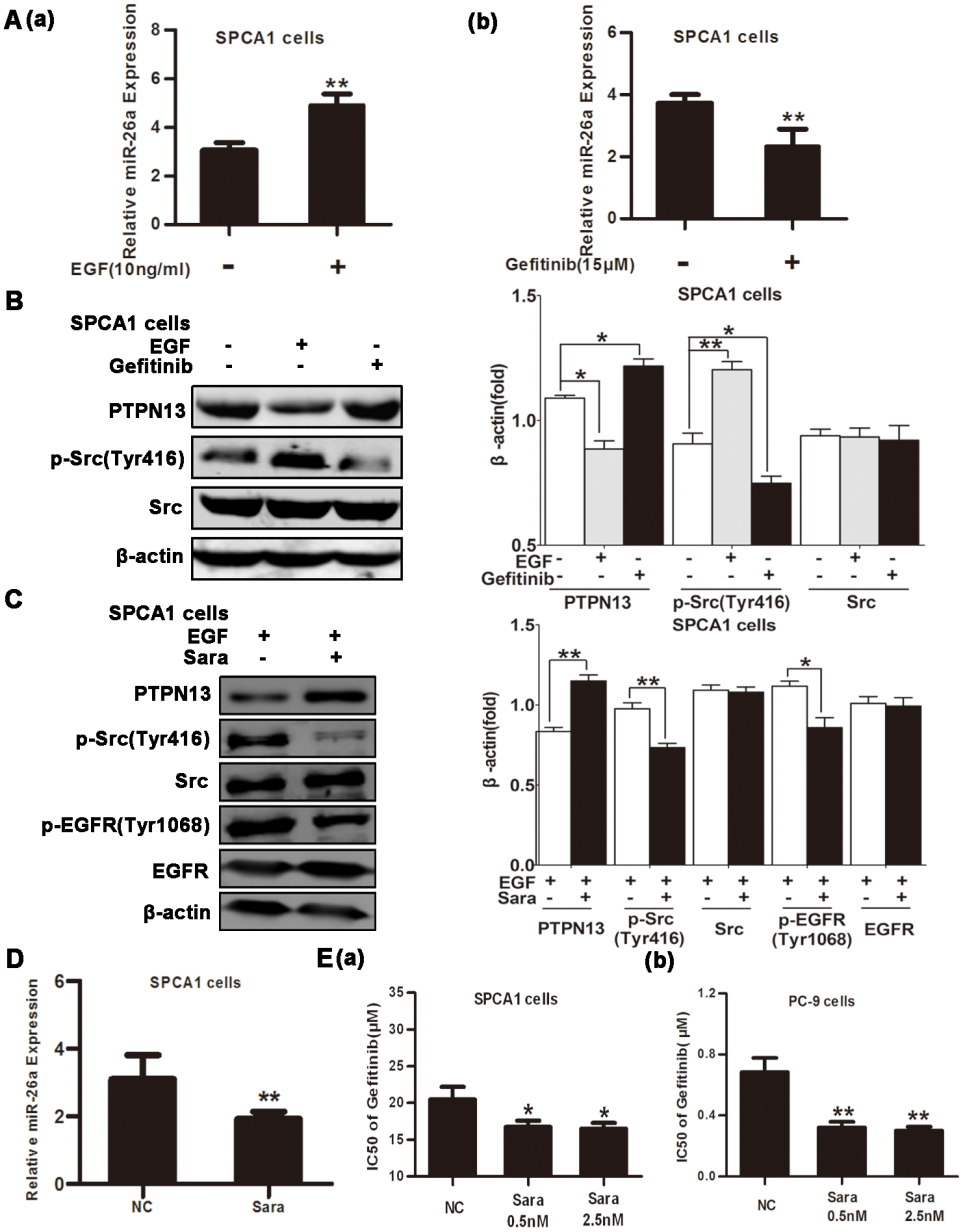
miR-26a is upregulated by EGFR/Src signaling **A.** qRT-PCR assays of SPCA1 cells after 48h incubation with EGF (10 ng/ml) (a) or 15 μM Gefitinib (b). Data are represented as the mean ± SD of three replicates. ** *P*<0.01, *vs* control group. **B.** Western blot assays of SPCA1 cells after treatment with EGF (10 ng/ml) or Gefitinib (15 μM). **C.** Western blot assays of SPCA1 cells after treatment with EGF (10 ng/ml) and with or without the Src inhibitor Saracatinib (Sara, 0.5 nM). **D.** qRT-PCR assays of SPCA1 cells after 48h incubation with or without Sara (0.5 nM) to detect miR-26a expression. Data are represented as the mean ± SD of three replicates. *P<0.05, ** *P*<0.01, *vs* control group. **E.** The IC50 values of Gefitinib for Sara-treated or control cells were measured and plotted as described in Figure 2B and 2D in SPCA1 cells (a) and PC-9 cells (b). Data are represented as the mean ± SD of three replicates. *P<0.05, ** *P*<0.01, *vs* control group. In western blot, data are representative of 3 independent experiments. All the protein levels measured with densitometry and normalized to β-actin. Each bar represents the mean±SD from three experiments.*p<0.05, **p<0.01*vs* control group.

The involvement of miR-26a in cancer cell resistance to EGFR-targeted TKIs may prompt a possible regulation of miR-26a expression by EGFR signal pathway. Consistent with this corollary, we found that EGF treatment of SPCA1 cells increased miR-26a expression and decreased PTPN13 protein levels(Figure 5A(a) & 5B), whereas gefitinib treatment of SPCA1 cells caused a significant downregulation of miR-26a levels and upregulation of PTPN13 protein levels (Figure 5A(b) & 5B), suggesting that EGFR signaling is at least partially involved in miR-26a expression in lung cancer cells. The interaction and reciprocal activation between EGFR and Src are critically involved in EGFR signaling. Consistently, we found that a Src inhibitor impeded the phosphorylation of both Src and EGFR (Figure 5C). Src inhibition caused a profound downregulation of miR-26a (Figure 5D) corresponding to upregulation of PTPN13 (Figure 5C) and concurrently an improved gefitinib responsiveness of lung cancer cells in SPCA1 cells (Figure 5E(a)) and PC-9cells (Figure 5E(b)). Taken together, miR-26a expression is increased by EGFR/Src signaling, thus forming a regulatory circuit to regulate the gefitinib sensitivity of NSCLC cells.

### miR-26a suppression sensitizes *in vivo* lung carcinomas to EGFR-TKIs

**Figure 6:**
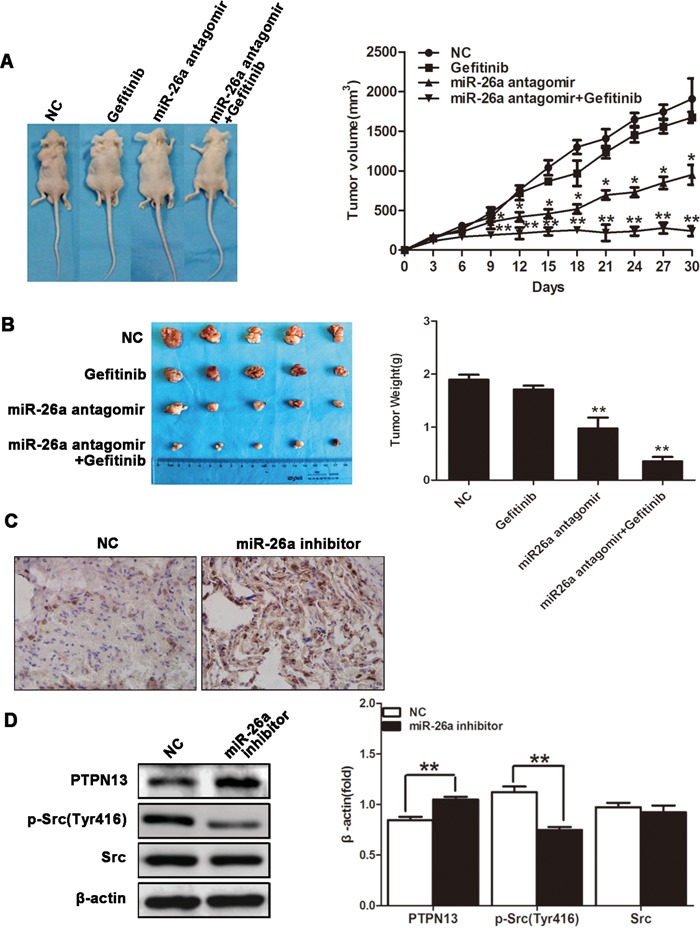
miR-26a confers Gefitinib resistance of xenograft NSCLCs Xenograft lung carcinoma was developed by s.c. challenging nude mice with SPCA1 cells. Mice then received gefitinib (25 mg/kg, daily) via oral gavage in combination with miR-26a antagomir (15 μg, twice a week) via intratumor injection (n=5). Tumor volume was monitored from the first day of gefitinib treatment **A.** Mice were sacrificed 30 d after the first injection of gefitinib, and tumors were dissected and weighed **B.**, followed by sectioning and immunohistochemical staining for PTPN13 in the tumor tissues **C.** Data are represented as the mean ± SD (n=5). *P<0.05, ** *P*<0.01, *vs* control group. Western blot assay was used to examine the protein expression level of PTPN13 and phospho-Src in tumor samples **D.** Data are representative of 3 independent experiments. All the protein levels measured with densitometry and normalized to β-actin. Each bar represents the mean±SD from three experiments. **p<0.01*vs* control.

We finally evaluated the role of miR-26a in the responsiveness of *in vivo* NSCLCs to TKI treatment. Nude mice were challenged with SPCA1 cells to allow the formation of subcutaneous tumors, followed by treatment with gefitinib by gavage and/or intratumor injection with miR-26a inhibitors. Compared with gefitinib treatment alone, gefitinib in combination with miR-26a inhibition showed significantly enhanced suppression on tumor growth (Figure 6A & 6B). The expression level of PTPN13 increased significantly in the tumor samples treated with miR-26a inhibitors by IHC study (Figure 6C). Consistent with the observations in cultured lung cancer cells, the administration of miR-26a inhibitors caused increased PTPN13 protein levels and decreased phospho-Src levels in NSCLC xenograft tumors (Figure 6D). Therefore, miR-26a plays an essential role in promoting the growth and EGFR-TKI resistance of *in vivo* NSCLCs.

## DISCUSSION

NSCLCs are the leading cause of cancer mortality worldwide, and are in urgent need of novel therapeutic strategies due to their relative insensitivity to radiation and chemotherapy [43]. The high incidence of EGFR tyrosine kinase domain mutation in NSCLCs has provided the rationale for successful application of a class of EGFR-TKIs for first-line lung adenocarcinoma treatment [44–45]. However, resistance of NSCLCs to EGFR-TKIs has become the major limitation for its therapeutic efficacy for patients [45–47]. The data we present here suggest that miR-26a promotes EGFR-TKI resistance in NSCLCs, and that miR-26a maintains Src activation by directly targeting and silencing PTPN13, which subsequently activates EGFR and its downstream signaling.

The last dozen years has witnessed the recognition of critical involvement of miRNAs in regulating gene expression and cell behaviors such as transformation [8, 10]. Unlike most individual miRNAs which play definitive oncogenic or tumor suppressive roles, miR-26a seems to either positively or negatively regulate carcinogenesis dependent on cell types and intracellular signaling context [48–49]. The tumor suppressor, PTEN, is one of miR-26a targets, through which miR-26a promotes the development of glioma, leukemia and lung cancer [18–20]. In line with its oncogenic role during pulmonary tumorigenesis, we established here that miR-26a also targets PTPN13 to promote cell growth and EGFR-TKI resistance of NSCLC cells. The nonreceptor-type tyrosine phosphatase PTPN13 is reported as a tumor suppressor gene in NSCLCs [50]. It is frequently inactivated in NSCLC through the loss of either mRNA and protein expression or somatic mutation [50]. The loss of PTPN13 increases signaling from EGFR and HER2 [50]. PTPN13 expression was down-regulated in Lung squamous carcinoma, and was negatively correlated with the cancer grade and stage [51]. Loss of PTPN13 correlated with poor overall survival in lung cancer patients [52]. PTPN13 has been documented to dephosphorylate a growing list of oncogenic protein substrates including human epidermal growth factor receptor 2 (HER2), insulin receptor substrate 1 (IRS-1) and thyroid receptor-interacting protein 6 (TRIP6) [53–54]. While it remains elusive whether all these substrates are involved in PTPN13 suppression of gefitinib resistance, our findings highlighted the role of Src inactivation in PTPN13-mediated gefitinib sensitization of NSCLCs, consistent with the previous finding that PTPN13 functions in NSCLCs as a tumor suppressor whose loss increases signaling from EGFR and HER2 [50]. Given that an individual miRNA may repress multiple mRNAs, it is possible that other genes, e.g. PTEN, are simultaneously targeted by miR-26a, which synergizes with PTPN13 silencing to maintain survival and proliferation of NSCLC cells in the presence of EGFR-TKIs [8, 18]. Despite the different mechanisms underlying cancer resistance to EGFR-TKIs and chemotherapy, these findings are in agreement with a recent report that miR-26a is among the key non-coding RNAs that mediate lung cancer resistance to cisplatin [55].

The regulatory role of miR-26a in cancer resistance to EGFR-TKIs may necessarily suggest its involvement in EGFR signaling or in mediating a crosstalk of other signal pathways with EGFR signaling [7, 46]. EGFR is generally activated through stimulation with EGF and autophosphorylation of its cytoplasmic tail, however it can alternatively be activated by non-receptor tyrosine kinase Src, which is one of the EGFR downstream transducers [56]. Src can transactivate EGFR by phosphorylating tyrosine 845 (Y845), which may eventually induce EGFR receptor full activation [57]. Based on this molecular mechanism, TKIs targeting Src family kinases such as saracatinib and dasatinib have been used as therapeutic drugs for NSCLC [58]. We found here that PTPN13 silencing by miR-26a facilitated the activation of Src, therefore, it is likely that sustained Src activation compensates for the kinase activity of EGFR inhibited by EGFR-TKIs. Alternatively, Src may play a competitive role in TKI binding to and kinase inhibition of EGFR. Meanwhile, gefitinib induces a moderate suppression of miR-26a, suggesting that miR-26a is at least partially regulated by EGFR signaling. However, it is reasonable to infer that other cellular machineries are involved in miR-26a upregulation in NSCLCs, and are the major drivers of miR-26a expression in the context of TKI-treated NSCLC cells. Therefore, it is of particular importance to decipher whether the canonical mechanisms underlying EGFR-TKI resistance, e.g. EGFR mutation, alternative growth factor signaling, or constitutive downstream kinase activity, are responsible for miR-26a regulation [7, 46]. One of the candidate cellular events underlying miR-26a upregulation may be Hedgehog signaling, which is widely involved in TKI resistance of various cancers, although the correlation between miR-26a levels and Hedgehog signaling in NSCLC cells remains to be validated [59–60]. Nevertheless, our findings unraveled the regulatory roles of miRNA(s) in EGFR-TKI responsiveness of NSCLCs, and thus hold out great promise for miR-26a as a potential target for treatment of EGFR-TKI resistant NSCLCs.

## MATERIALS AND METHODS

### Cell culture and transfection

The immortalized bronchial epithelial cell line BEAS-2B and the human lung cancer cell lines A549, H520, SW900, H2170 and PC-9 were purchased from ATCC (Manassas, VA, USA). The human lung cancer SPCA1 cell line was obtained from the Cell Bank of Shanghai Institute for Biological Sciences, Chinese Academy of Sciences. All cell lines were characterized by gene profiling by the providers, and were propagated and frozen for future study after receipt. Each vial of frozen cells was thawed and maintained in culture for a maximum of 8 weeks. Enough frozen vials were available for each cell line to ensure that all cell-based experiments were conducted on cells that had been tested and in culture for 8 weeks or less.

Lung cancer cells were cultured in RPMI 1640 or DMEM medium supplemented with 10% fetal bovine serum (FBS) and maintained at 37°C in a humidified incubator with 5% CO_2_. The cultures were split at 90% confluence, and the media were changed every 2 days. Where indicated, cells were treated with Gefitinib (AstraZeneca, London, UK), human EGF (BD Biosciences, San Jose, USA) or the Src inhibitor Saracatinib (Selleckchem, Houston, USA).

The miR-26a mimics and antagomirs, and PTPN13-targeted siRNAs or mismatched control were purchased from Shanghai Genepharma Inc. (Shanghai, China). The following sequences of PTPN13 mRNA were targeted by siRNAs: 5′-GCAGTAACAGTGCGGACTT-3′ (siPTPN13-1), and 5′-CCTTATTGCTGGCATCCTT-3′ (siPTPN13-2). When cultures reached 50–60% confluence, transfection was performed using Lipofectamine 2000 reagent (Life Technologies, Grand Island, USA), according to the manufacturer's instructions at a final concentration of 30 nM (for miR-26a mimics) or 60 nM (for miR-26a antagomirs).

### MTT assay

Cells (3000/well) were seeded in 96-well plates in complete medium after overnight serum starvation. Transfection was performed as aforementioned. Cell proliferation was measured using the MTT [3-(4, 5-dimethylthiazol-2-yl)-2, 5-diphenyl-2*H*-tetrazolium bromide] assay. Briefly, 48 h after transfection, the transfection medium was replaced with 100 μL fresh serum-free medium with 0.5 g/L MTT. After incubation at 37°C for 4 h, the MTT-containing medium was removed by aspiration and 50 μL DMSO was added. After incubation at 37°C for an additional 10 min, absorbance was measured at 450 nm in a plate reader.

### Quantitative RT-PCR

NSCLC samples and the paired adjacent normal tissues were obtained from surgical specimens. EGFR genome status was examined by commercial next generation sequencing test. Tumor sample 4 had EGFR exon 20 insertion mutation, the other 4 tumor samples were EGFR wild type. All the five tumor samples were EGFR-TKI resistant.

To quantitatively determine the mRNA levels of miR-26a and PTPN13 in patient samples, NSCLC cell lines and immortalized bronchial epithelial cell line, BEAS-2B, quantitative RT-PCR was used. For RNA isolation, total RNA was extracted and isolated from tissue samples or cell lines using either the mirVana miRNA isolation kit (Ambion, Austin, TX) or the TRIzol method (Life Technologies) according to the manufacturer's protocol. Reverse transcription was performed using SuperScript™ II reverse transcriptase (Life Technologies), and cDNAs were amplified and detected using the SYBR Premix Ex Taq™ (TaKaRa, Dalian, China). To quantify miRNAs, total RNA was reverse-transcribed using the miScript Reverse Transcription Kit (Qiagen, Hilden, Germany) and then amplified using SYBR Premix Ex TaqTM (TaKaRa). β-actin and U6 RNA were used as internal loading controls for mRNAs and miRNAs, respectively. The primers used for PCR were as follows: 5′-GATCATTGCTCCTCCTGAGC-3′ and 5′-ACTCCTGCTTGCTGATCCAC-3′ for β-actin, 5′-GGATTTGCCACGGTCTATTCT-3′ and 5′-CAGGTCAGTTCCAGTCTTTGC-3′ for PTPN13, and universal primer (UP) in the miScript reverse transcription kit and 5′-TTCAAGTAATCCAGGATAGGCT-3′ or 5′-GTGCTCGCTTCGG CAGCACATAT-3′ for miR-26a or U6 RNA, respectively.

### Western blotting

Cells were harvested, and proteins were extracted, separated on an SDS/PAGE gel, transferred onto PVDF membranes and subjected to immunoblot analyses. Blotting was performed using antibodies targeting PTPN13 (Abcam, Cambridge, UK; Catalog #: ab10867), Akt (Cell Signaling, Danvers, USA; Cat. #: 9272), phosphorylated Akt (pAkt, Cell Signaling; Cat. #: 4060), ERK (Cell Signaling; Cat. #: 9102), pERK (Cell Signaling; Cat. #: 4370), Src (Cell Signaling; Cat. #: 2109), pSrc (Y416, Cell Signaling; Cat. #: 2101), and β-actin (Sigma-Aldrich; Cat. #: SAB5500001).

### Luciferase reporter assay

The 3′UTR of PTPN13 was amplified and cloned from the lung epithelial BEAS-2B cells using primers 5′-CGGAATTCGCCTCTGGATGCATTTCCAT-3′ and 5′-AAAACTGCAGGTCT GCACACAGTTTTGGCT-3′, and a variant containing mutations in the putative miR-26a binding site was generated using a Multipoints Mutagenesis Kit (Takara, Catalog #: R407). The wild-type and mutated 3′UTR were inserted downstream of the firefly luciferase gene in the pGL3 vector (Promega, Madison, USA). HEK293 cells were co-transfected with reporter constructs, an internal control vector (pGL4.73), and a synthetic miR-26a mimic. 48 h after transfection, luciferase activity was assayed using the Dual-Luciferase Reporter Assay System (Promega) and a luminometer (Glomax 20/20, Promega), and normalized to the activity of Renilla luciferase driven by a constitutively expressed promoter in the phRL vector. Basal promoter activity was measured as the fold change relative to the activity observed with the basic pGL3 vector alone.

### Immunoprecipitation

Cells were lysed in buffer containing 1% (v/v) Nonidet P-40, 0.5 mM EGTA, 5 mM sodium orthovanadate, 10% (v/v) glycerol, 100 μg/mL phenylmethylsulfonyl fluoride, 1 μg/mL leupeptin, 1 μg/mL pepstatin A, 1 μg/mL aprotinin and 50 mM HEPES, pH 7.5. Aliquots of 500 μL diluted lysate (1μg protein/μL) were incubated overnight with 5 μL antibody against Src. The immune complex was captured by adding 80 μL of a 1:1 (v/v) protein A-Sepharose 4B bead suspension and incubating the mixture for an additional 90 min. The beads were harvested and the proteins bound to them were resolved by SDS-PAGE and analyzed by Western blot. The primary antibodies used for immunoprecipitation are described in the Western blotting methods.

### Modeling studies

In accord to the previous reports [21–22], the coordinates of PTPN13 (accession number: NP_542416.1) protein was built by the MODELER module in Insight II 2005 software packages [23]. The initial structure of Src kinase was retrieved from the RCSB Protein Data Bank (entry code 2SRC) [24], and its phosphorylation state (Src^pTyr416^) was generated by replacing the corresponding amino acid (Tyr → pTyr). The two generated models were equilibrated by MD simulations using GROMACS4.6.7 program [25] and Charmm27 force field [26], as previously recommended [22, 27]. The docking simulations were performed using the ZDOCK and RDOCK modules within Insight II 2005 [28–30]. The optimal docked complex was selected on basis of energy and the size of cluster, and then optimized using the conjugated gradient (CG) algorithm, with a convergence criterion of 0.01 kcal·mol-1·Å-1. The energy-minimized docked complex was equilibrated by 200.0 ns MD simulations, using GROMACS4.6.7 program [25] and Charmm27 force field [26]. Details of the MD simulation setup are in agreement with reference [31].

### *In vivo* tumor xenograft mouse model

BALB/c nude mice (Institute of Zoology, Chinese Academy of Sciences) that were 4-6 weeks old were challenged subcutaneously with 1×10^7^ lung cancer SPCA1 cells to allow tumor development. When palpable tumors (approximately 100 mm^3^) arose, the mice in each group were randomly grouped and treated intratumorally with miR-26a or control antagomir (15μg/injection, twice a week), and/or with gefitinib (25mg/kg/d) by oral gavage. Tumor growth was monitored by caliper measurements of the two perpendicular diameters every 3 days, and the volume of the tumor was calculated with the formula V = (width^2^×length/2). All procedures were performed in compliance with the Regulations for the Administration of Affairs Concerning Experimental Animals (approved by the State Council of the People's Republic of China) and were approved by the Experimental Animal Ethics Committee of The Fourth Military Medical University.

### Immunohistochemistry

Xenograft tumors were isolated from tumor-bearing mice, and a set of 4-μm-thick paraffin sections were cut and mounted on silanized slides. Sections were subjected to immunohistochemial staining via a standard 3-step immunoperoxidase protocol using anti-human PTPN13 monoclonal Ab (Abcam; Cat. #: ab49446; dilution: 1:40), and control staining was performed using isotype rabbit IgG (Cell Signaling).

### Statistical analysis

Statistical significance was assessed by comparing mean (± SD) values with Student's *t*-test for independent groups. *P* ≤ 0.05 was considered statistically significant.

### Summary

In this study, we found that miR-26a is overexpressed in EGFR-TKI resistant NSCLC cells, and that miR-26a confers EGFR-TKI resistance of NSCLC cells by targeting and silencing PTPN13; PTPN13 downregulation increases Src activation and enhances EGFR downstream signaling.

## SUPPLEMENTARY FIGURES



## Abbreviations

EGFR: epidermal growth factor receptor
HER: human epidermal growth factor receptor
NSCLC: non-small cell lung cancer
TKI: tyrosine kinase inhibitor
PTPN13: protein tyrosine phosphatase non-receptor type 13
UTR: untranslated region.
