# Supplementary material for: miR-26a desensitizes non-small cell lung cancer cells to tyrosine kinase inhibitors by targeting PTPN13
Source: Oncotarget. 2016 Jun 7;7(29):45687–701. doi: 10.18632/oncotarget.9920 (PMC5216753; doi:10.18632/oncotarget.9920)
Supplement: Supplementary file 1 [file oncotarget-07-45687-s001.pdf]

miR-26a desensitizes non-small cell lung cancer cells to tyrosine kinase inhibitors by targeting PTPN13

SUPPLEMENTARY FIGURES

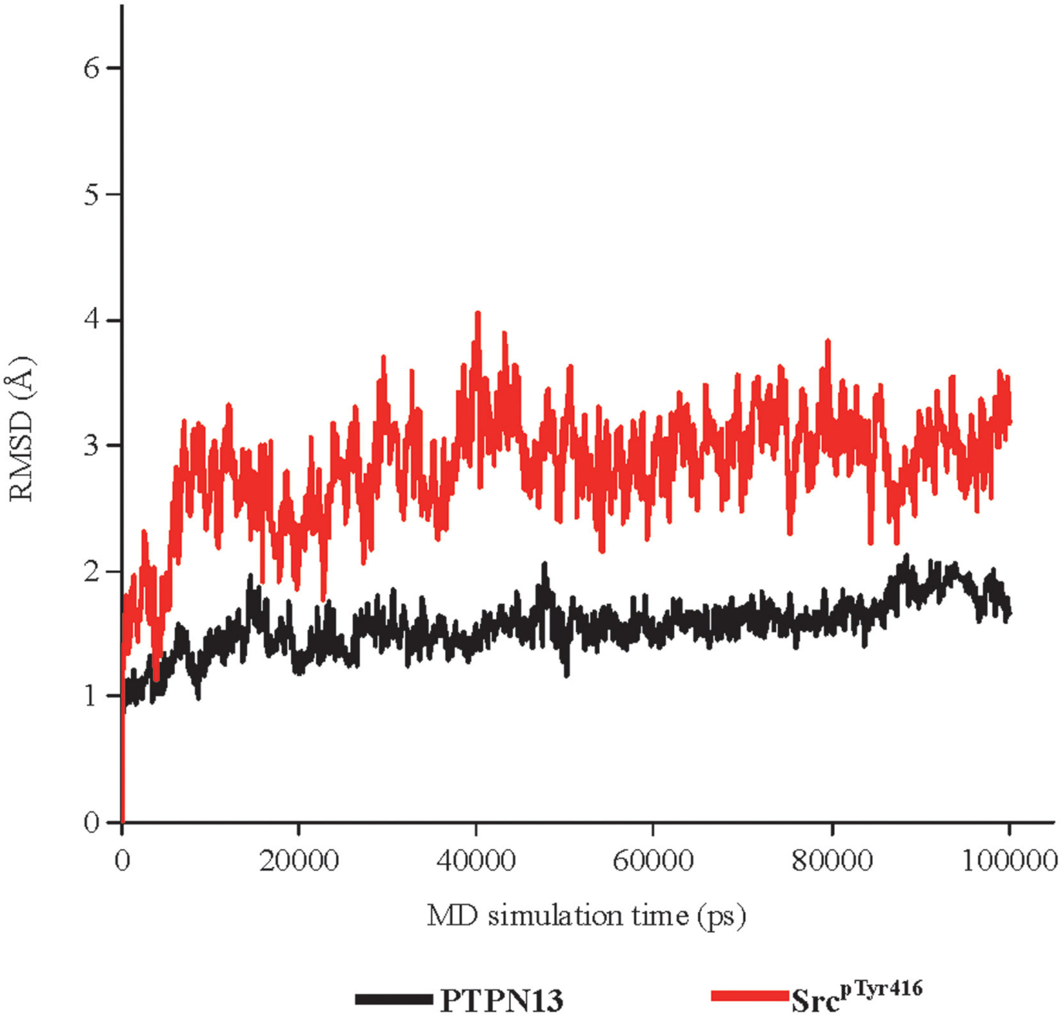

Supplementary Figure S1: Backbone-atom RMSD of PTPN13 (in black) and Src (in red) with respect to simulation time (100 ns).

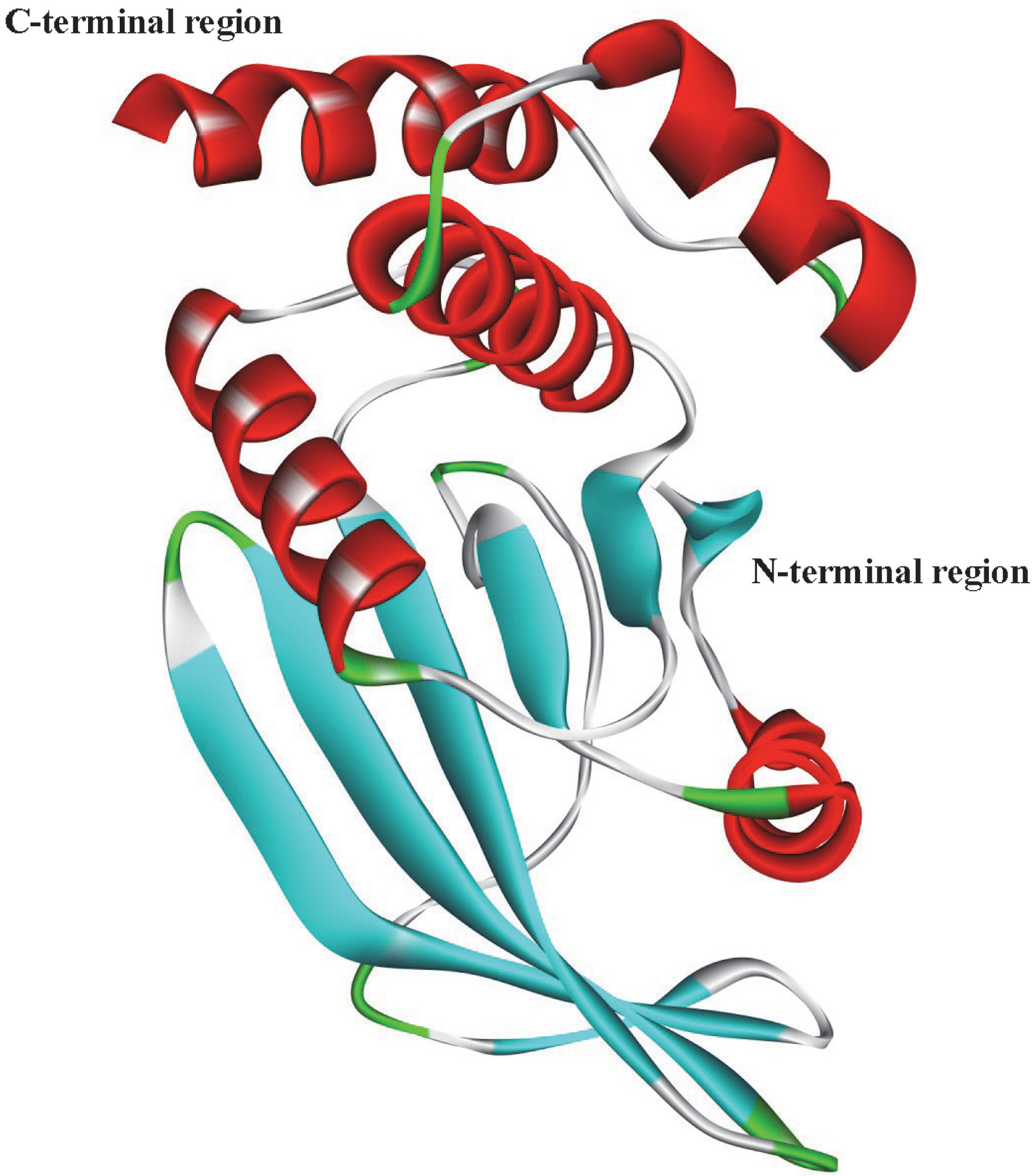

Supplementary Figure S2: Homology modeling structure of PTPN13 (AA 2277-2470) after the 100-ns MD simulation. Helices are red, beta sheets are cyan, turns are green, and coils are white.

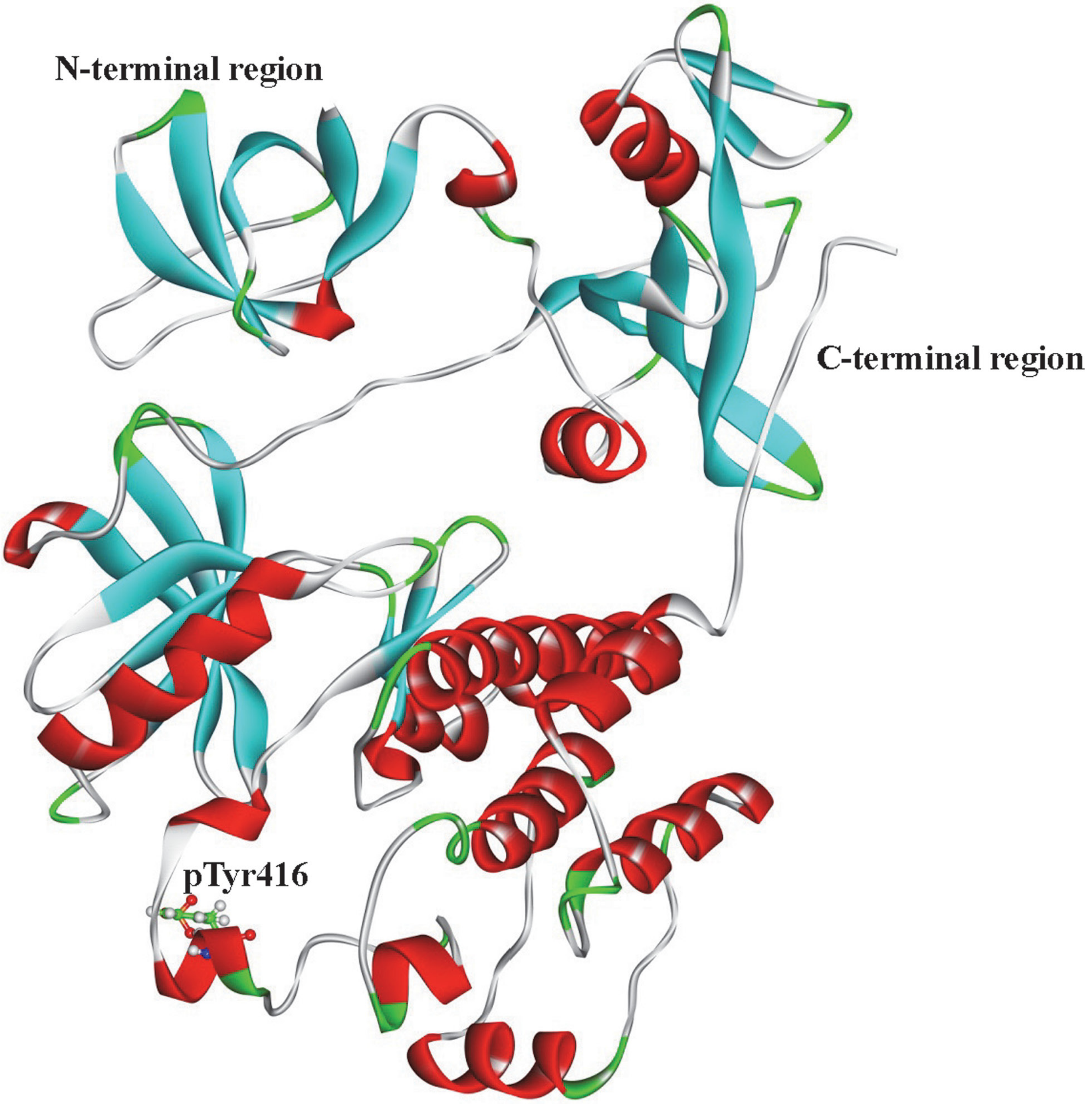

Supplementary Figure S3: Structure of tyrosine-phosphorylated Src (Src^{pTyr416}) after the 100-ns MD simulation. Helices are red, beta sheets are cyan, turns are green, and coils are white. Phosphorylation site is represented by ball and stick model.

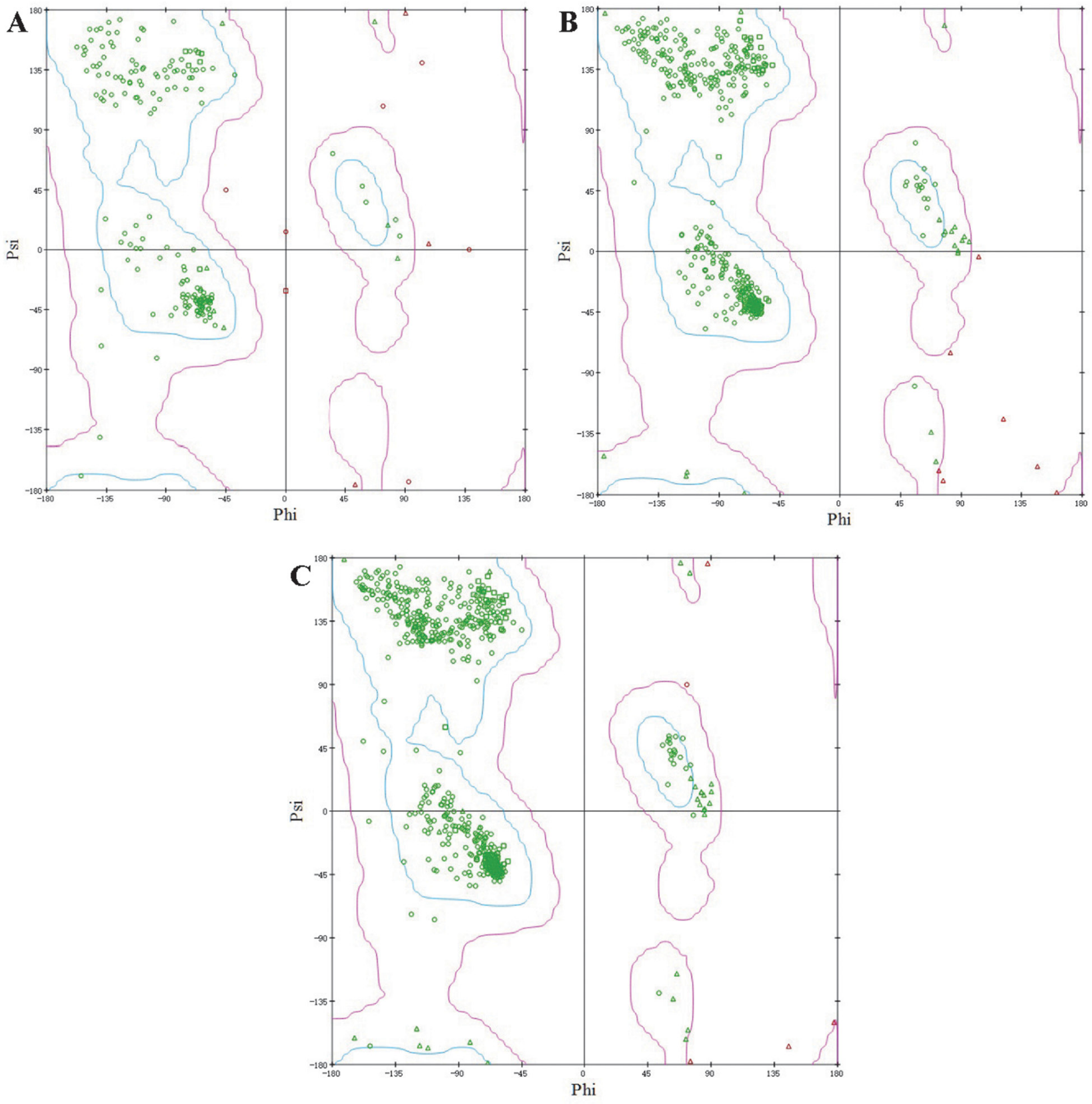

Supplementary Figure S4: Ramachandran plots of A. PTPN13, B. Src^{pTyr416} and C. PTPN13-Src^{pTyr416} after the MD simulations. Ramachandran plot shows phi-psi torsion angles of all residues in the structure. The coloring/shading on the plot represents the different regions: the darkest areas (shown here in red) correspond to the "core" regions representing the most favorable combinations of phi-psi values. The percentage of residues in the "core" regions is one of the better indications of stereochemical quality.
